# Supplementary material for: Allosteric ligands control the activation of a class C GPCR heterodimer by acting at the transmembrane interface
Source: eLife. 2021 Dec 6;10:e70188. doi: 10.7554/eLife.70188 (PMC8700296; doi:10.7554/eLife.70188)
Supplement: Supplementary file 1. — Data represent the means ± SEM of (n) independent experiments. ****p<0.0001 (one-way ANOVA test); N.A., not applicable; nH, Hill slope. [file elife-70188-supp1.docx]

**Supplementary File 1.** Allosteric agonist activity of the PAMs on the wild-type GABA_B_ receptor. Intracellular Ca^2+^ responses mediated by GABA and the indicated PAMs. Data represent the means ± SEM of (n) independent experiments. **** *P* < 0.0001 (one-way *ANOVA* test); N.A.: not applicable; n_H_: Hill slope.

|  | pEC_50_ | n_H_ | Emax (% of max GABA response) |
| --- | --- | --- | --- |
| GABA | 7.39 ± 0.03 (16) | 0.77 ± 0.03 (16) | 97.97 ± 0.61 (16) |
| rac-BHFF | 4.79 ± 0.03 (15) **** | 1.74 ± 0.18 (15) | 68.91 ± 2.44 (15) **** |
| CGP7930 | 4.45 ± 0.17 (4) **** | 3.59 ± 6.07 (4) | 50.98 ± 9.82 (4) **** |
| GS39783 | N.A. | N.A. | N.A. |
