# Supplementary material for: Allosteric ligands control the activation of a class C GPCR heterodimer by acting at the transmembrane interface
Source: eLife. 2021 Dec 6;10:e70188. doi: 10.7554/eLife.70188 (PMC8700296; doi:10.7554/eLife.70188)
Supplement: Supplementary file 2. — Intracellular Ca2+ responses mediated by the indicated constructs upon stimulation with GABA in the absence or presence of the indicated concentrations of a PAM. Data represent the means ± SEM of (n) independent experiments. *p<0.05, **p<0.005, ***p<0.0005, ****p<0.0001 (one-way ANOVA test); nH, Hill slope. [file elife-70188-supp2.docx]

**Supplementary File 2.** Allosteric modulation of the PAMs on the indicated GABA_B_ receptor constructs. Intracellular Ca^2+^ responses mediated by the indicated constructs upon stimulation with GABA in the absence or presence of the indicated concentrations of a PAM. Data represent the means ± SEM of (n) independent experiments. * *P* < 0.05, ** *P* < 0.005, *** *P* < 0.0005, **** *P* < 0.0001 (one-way *ANOVA* test); n_H_: Hill slope.

| Receptor | Ligand | pEC_50_ | n_H_ | Emax (% of max GABA response) |
| --- | --- | --- | --- | --- |
| GB1  +  GB2 | GABA | 7.45 ± 0.02 (14) | 0.79 ± 0.02 (14) | 97.90 ± 0.43 (14) |
|  | +rac-BHFF 10 μM | 7.52 ± 0.07 (5) | 0.69 ± 0.07 (5) | 101.80 ± 1.33 (5) * |
|  | 20 μM | 7.47 ± 0.10 (4) | 0.57 ± 0.06 (4) * | 105.00 ± 1.48 (4) **** |
|  | +CGP7930 10 μM | 7.67 ± 0.04 (5) ** | 0.77 ± 0.05 (5) | 100.80 ± 1.02 (5) |
|  | 20 μM | 7.82 ± 0.07 (4) **** | 0.76 ± 0.07 (4) | 101.90 ± 1.39 (4) * |
|  | +GS39783 10 μM | 7.83 ± 0.06 (4) **** | 0.79 ± 0.07 (4) | 100.40 ± 1.30 (4) |
|  | 20 μM | 7.97 ± 0.09 (3) **** | 0.79 ± 0.11 (3) | 101.30 ± 1.98 (3) |
|  |  |  |  |  |
| GB1  +  M14 | GABA | 6.42 ± 0.05 (12) | 0.85 ± 0.07 (12) | 99.70 ± 1.48 (12) |
|  | +rac-BHFF 10 μM | 6.72 ± 0.09 (6) ** | 0.64 ± 0.08 (6) | 107.40 ± 2.73 (6) * |
|  | 20 μM | 7.13 ± 0.09 (6) **** | 0.60 ± 0.07 (6) | 110.80 ± 2.42 (6) *** |
|  | +CGP7930 10 μM | 6.69 ± 0.04 (2) | 0.87 ± 0.05 (2) | 101.60 ± 1.13 (2) |
|  | 20 μM | 6.89 ± 0.06 (2) ** | 0.84 ± 0.09 (2) | 106.60 ± 1.82 (2) |
|  | +GS39783 10 μM | 6.73 ± 0.05 (4) * | 0.92 ± 0.09 (4) | 103.80 ± 1.68 (4) |
|  | 20 μM | 6.89 ± 0.06 (4) *** | 0.76 ± 0.07 (4) | 109.60 ± 1.86 (4) * |
|  |  |  |  |  |
| GB1  +  M15 | GABA | 6.58 ± 0.06 (12) | 0.77 ± 0.07 (12) | 98.27 ± 1.73 (12) |
|  | +rac-BHFF 10 μM | 6.65 ± 0.08 (6) | 0.68 ± 0.08 (6) | 101.50 ± 2.36 (6) |
|  | 20 μM | 7.00 ± 0.08 (6) *** | 0.67 ± 0.08 (6) | 104.70 ± 2.21 (6) |
|  | +CGP7930 10 μM | 7.12 ± 0.05 (2) ** | 0.98 ± 0.11 (2) | 101.00 ± 1.55 (2) |
|  | 20 μM | 7.23 ± 0.09 (2) *** | 0.85 ± 0.13 (2) | 110.30 ± 2.51 (2) * |
|  | +GS39783 10 μM | 7.01 ± 0.05 (4) ** | 0.80 ± 0.07 (4) | 101.20 ± 1.41 (4) |
|  | 20 μM | 7.15 ± 0.07 (4) **** | 0.67 ± 0.07 (4) | 108.20 ± 1.92 (4) * |
