# Supplementary material for: Allosteric ligands control the activation of a class C GPCR heterodimer by acting at the transmembrane interface
Source: eLife. 2021 Dec 6;10:e70188. doi: 10.7554/eLife.70188 (PMC8700296; doi:10.7554/eLife.70188)
Supplement: Supplementary file 3. — Intracellular Ca2+ responses mediated by the indicated constructs upon stimulation with rac-BHFF. Data represent the means ± SEM of (n) independent experiments. ***p<0.0005, ****p<0.0001 (one-way ANOVA test); N.A., not applicable; nH, Hill slope. [file elife-70188-supp3.docx]

**Supplementary File 3.** Allosteric agonist activity of rac-BHFF on the indicated GABA_B_ receptor constructs. Intracellular Ca^2+^ responses mediated by the indicated constructs upon stimulation with rac-BHFF. Data represent the means ± SEM of (n) independent experiments. *** *P* < 0.0005, **** *P* < 0.0001 (one-way *ANOVA* test); N.A.: not applicable; n_H_: Hill slope.

|  | pEC_50_ | n_H_ | Emax (% of control) |
| --- | --- | --- | --- |
| GB1+GB2 | 4.78 ± 0.02 (21) | 1.62 ± 0.09 (21) | 105.10 ± 2.11 (21) |
| GB1+GB2^ΔECD^ | 4.84 ± 0.07 (5) | 2.03 ± 0.54 (5) | 78.09 ± 5.88 (5) *** |
| GB1^ΔECD^+GB2^ΔECD^ | 4.75 ± 0.10 (3) | 1.77 ± 0.59 (3) | 51.84 ± 5.93 (3) **** |
| GB1^ΔECD^+GB2 | 4.80 ± 0.08 (8) | 1.54 ± 0.38 (8) | 66.59 ± 5.78 (8) **** |
| GB^2/1^+GB2 | 4.74 ± 0.05 (5) | 1.36 ± 0.17 (5) | 111.00 ± 5.80 (5) |
| GB^1/2^+GB2 | 6.68 ± 0.64 (4) **** | 1.00 ± 1.43 (4) | 1.64 ± 0.32 (4) **** |
| GB1^TM7^+GB2 | N.A. | N.A. | N.A. |
| GB1^ASA^ | N.A. | N.A. | N.A. |
| GB2 | N.A. | N.A. | N.A. |
